# A randomized controlled trial to assess parental satisfaction with computerized intraosseous anesthesia versus inferior alveolar nerve block in children

**DOI:** 10.1038/s41598-024-66359-5

**Published:** 2024-07-04

**Authors:** Muaaz Alkhouli, Zuhair Al-Nerabieah, Mayssoon Dashash

**Affiliations:** Paediatric dentistry department, Faculty of Dentistry, Damascus University, Damascus, Syria

**Keywords:** Parental satisfaction, Children, Local anesthesia scale, PSLAS scale, Computerized intraosseous anesthesia, Paediatric research, Clinical trial design, Health care, Medical research

## Abstract

This study aimed to compare parental satisfaction between two pediatric dental anesthesia techniques, computerized intraosseous anesthesia (CIA) and inferior alveolar nerve block (IANB). This study was designed as a split-mouth randomized controlled clinical trial. A total of 52 parents of children undergoing dental treatment were enrolled in the study. Each participant received both CIA and IANB anesthesia, with the order of administration randomized. Parental satisfaction was evaluated using the parental satisfaction of dental local anesthetic techniques scale (PSLAS). Statistical analysis revealed that parental satisfaction regarding CIA was higher than that for IANB with a significant difference (*P* ˂ 0.05). However, there was no difference regarding the age, gender or the education level of the parents. (*P* > 0.05). This study provides insights into parental satisfaction with pediatric dental anesthesia techniques and highlights the influence of socioeconomic factors on anesthesia decision-making. Within the limitations of this trial, it was concluded that CIA was significantly superior to IANB in overall parental satisfaction. However, parental satisfaction values were lower in CIA group regarding costs and concern from complications. In addition, it was concluded that there was no difference in satisfaction levels regarding the gender, age and education level of the parents.

## Introduction

Managing of anxiety and discomfort associated with dental procedures remains a significant challenge, particularly among younger patients, despite advancements in pediatric dental care^1^. Anxiety related to dental visits can have long-lasting effects, leading to dental phobia and avoidance behaviors later in life^2^. Thus, effective pain management strategies, such as local anesthesia techniques, are paramount in promoting positive dental experiences and mitigating the risk of dental anxiety development^3^.

In recent years, there has been a growing emphasis on patient-centered care within the field of dentistry, particularly in pediatric settings^4^. Recognizing the unique needs and preferences of pediatric patients and their families, dental professionals are increasingly prioritizing interventions that not only ensure clinical efficacy but also optimize patient comfort and satisfaction^5^. Anesthesia techniques play a pivotal role in this regard, as they directly influence the patient’s experience during dental procedures and can significantly impact overall satisfaction with the treatment process^6^.

Local anesthesia is an essential part of pediatric dentistry, facilitating painless procedures and minimizing the necessity for sedation or general anesthesia^7^. However, conventional techniques like the inferior alveolar nerve block (IANB) are often beset by limitations such as injection pain, collateral anesthesia, postoperative complications, and diminished acceptance by both children and their parents^8^.

In response to these challenges, the computerized intraosseous anesthesia technique (CIA) emerges as a novel approach to administering local anesthesia^9^. CIA utilizes a computer-controlled device to deliver anesthetic solutions to dental and peri-dental tissues by penetrating the bone^10^. This technique is purported to offer several advantages over traditional methods, including expedited onset, shortened duration, heightened success rates, and reduced discomfort^11^.

Among the notable implementations of CIA is the Quicksleeper 5, which employs a high-speed rotating needle to create micro-ostectomies in cortical bone and administer anesthetic solutions into cancellous bone. Extensive research corroborates the efficacy and safety of Quicksleeper 5 across various pediatric dental procedures such as pulpotomy, pulpectomy, and extractions^12,13^.

In the realm of pediatric dentistry, parental satisfaction serves as a crucial metric reflecting both the quality of care and the patient’s overall experience. Numerous factors can influence parental satisfaction, including the child’s pain perception, behavioral response, personal preferences, and postoperative outcomes^14–16^. Hence, evaluating parental satisfaction becomes imperative in assessing the effectiveness and acceptability of different anesthesia techniques.

Moreover, the influence of parental attitudes and perceptions on pediatric dental care cannot be overstated. Parents play a crucial role in decision-making processes regarding their child’s dental treatment, including the choice of anesthesia technique. Understanding parental preferences, concerns, and satisfaction levels is integral to providing patient-centered care and fostering collaborative relationships between dental professionals, children, and their families^17,18^.

Additionally, exploring parental satisfaction with different anesthesia techniques not only sheds light on the efficacy and acceptability of these methods but also informs evidence-based practice guidelines and clinical decision-making.

Therefore, the aim of this study is to compare parental satisfaction regarding the use of Quicksleeper 5 and IANB for local anesthesia in children undergoing dental treatment. The null hypothesis posits that there is no difference in parental satisfaction between the two techniques. The alternative hypothesis suggests that Quicksleeper 5 is superior to IANB in terms of parental satisfaction.

## Methods

### Study design

This study is a prospective split-mouth single-blinded randomized controlled clinical trial that compares the parental satisfaction regarding the use of computerized Intraosseous anesthesia (CIA) and the use of inferior alveolar nerve block (IANB) for local anesthesia in children undergoing dental treatment. The study was adopted according to the consolidated standards of reporting trials (CONSORT) statement criteria to ensure rigorous and transparent reporting of the study methodology and results. In addition, it was conducted in accordance with the declaration of Helsinki, authorized by the ethical committee of the faculty of Dentistry at Damascus University on 22-07-2021 (3662) and registered at the International Standard Randomised Controlled Trial Number Registry (ISRCTN) (trial id: ISRCTN60155644) on 12-1-2022. The present study was performed during the period between December 2022 to June 2023.

### Study participants

Participants were recruited from the pediatric dental department at Damascus University. The study sample consists of 52 parents who have children aged 7–9 years and required bilateral pulpotomy for mandibular primary second molars. The inclusion criteria were: (1) diagnosis of reversible pulpitis confirmed by clinical and radiographic examination, (2) no history of allergy or contraindication to lidocaine or epinephrine, (3) no systemic disease or medication that may affect pain perception. All children who were unable to cooperate with the study procedure were excluded. In addition, children who have infection in the area of anesthetic injection were also excluded from this study.

### Sample size determination

The sample size was calculated using G*Power software version 3.1.9.4. Based on the results of a previous study^19^, the expected mean difference in the parental satisfaction of dental local anesthetic techniques scale (PSLAS) scores between the two groups was estimated to be 2.1 with a standard deviation of 3.7. The power of the study was set at 90%, with an alpha level of 0.05. The final sample size was 52 children. Hence, 104 questionnaires were analyzed.

### Study groups and randomization

Group A (control group): parental satisfaction was studied after their children have experienced inferior alveolar nerve block (IANB), n = 52.

Group B (intervention group): parental satisfaction was studied after the using of computerized intraosseous anesthesia using QuickSleeper 5 (Dental hi tech, France), n = 52.

**Figure 1:**
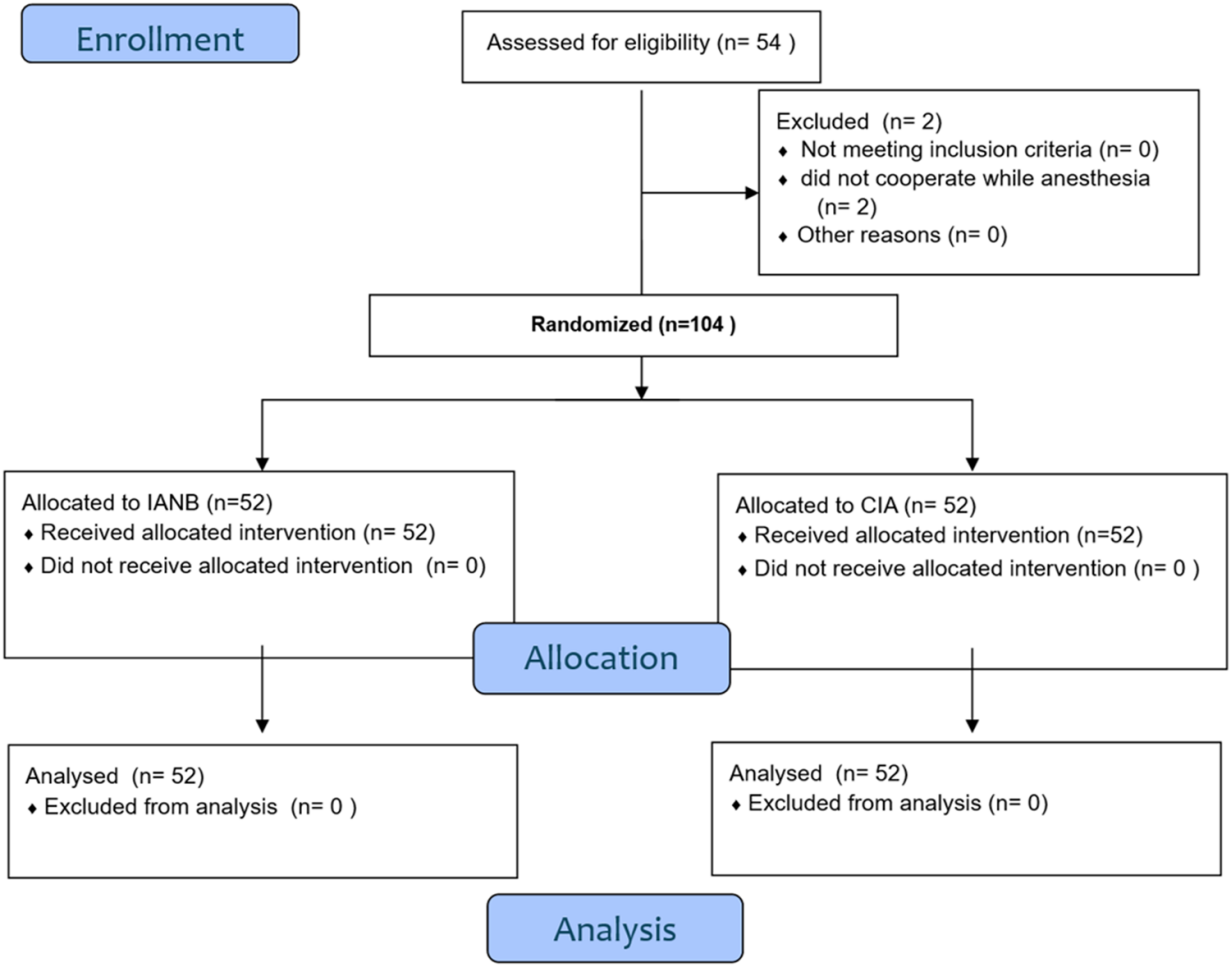
Trial consort flow chart.

In this split-mouth study, randomization was performed based on two factors: the type of anesthetic technique (CIA/IANB) and the side of the mouth (left/right). Each participant drew twice from opaque envelopes. The first draw determined the anesthetic technique to be applied first, and the second draw determined the side to be treated with the selected technique. Figure 1 shows the consort flow diagram of the progress through this randomized trial.

### Intervention

**Figure 2:**
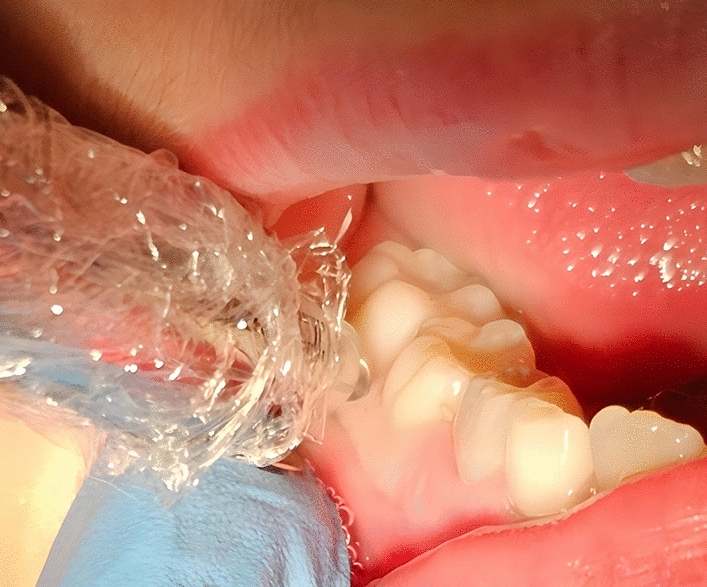
The technique of anesthesia in the CIA group.

Children in the CIA group received local anesthesia using the Quicksleeper 5 device (DHT, France), which consists of a handpiece, a needle, and a control unit. The needles used are of 9.0 mm length and 27-gauge diameter. The device delivers the anesthetic solution into the cancellous bone through a high-speed rotating needle that creates a micro-ostectomy in the cortical bone. After drilling the bone, the device injected 0.9 ml of 2% Lidocaine with 1:200,000 epinephrine (Fig. 2). In the CIA group, we deliverd the anesthetic solution between the permanent lower first molar and the primary lower second molar. In this manner, all of pulpal, bone and soft tissue of the tooth mesial and distal to the injection point will be anesthetized according to the review of Tom et al.^9^. Therefore, Inferior Alveolar nerve, lingual and buccal nerves were anesthetized. Therefore, there were two punctures; one was for gingival anesthesia and the second was for the intraosseous anesthesia.

**Figure 3:**
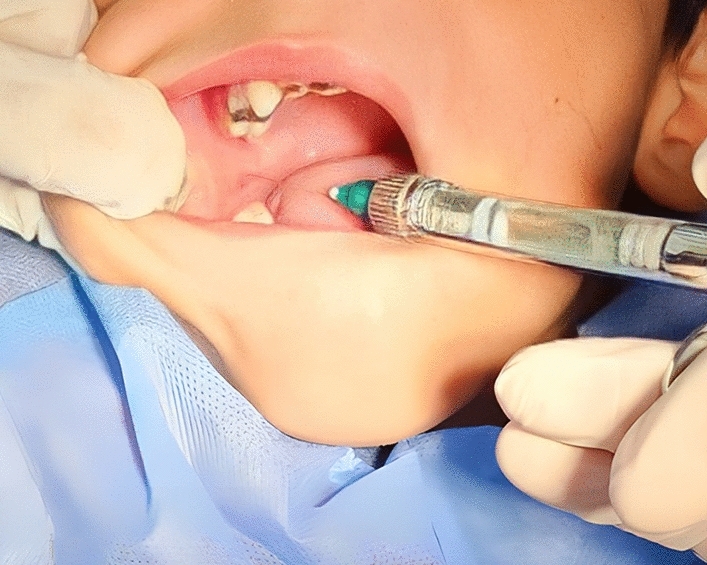
The technique of anesthesia in the IANB group.

On the other hand, children in IANB group received local anesthesia using a conventional syringe and a 27-gauge needle. The anesthetic solution and the quantity injected were the same as the CIA group. The injection site was near the mandibular foramen, which was detected by palpating the coronoid notch and the pterygomandibular raphe. The needle was inserted until the bone was contacted, and then retracted 1 mm. The anesthetic solution was injected slowly until 0.9 ml was delivered (Fig. 3). Inferior alveolar nerve and lingual nerves were anesthetized during the block technique. However, we performed an infiltration anesthesia for the buccal side of the lower permanent 1st molar to anesthetize the nerve endings of the buccal nerve as we placed a clump on the this molar during rubber dam placement. Therefore, In the IANB technique, there was one puncture point during the block technique and the other was for buccal nerve anesthesia.

Structured interviews were conducted with parents to gather data regarding their satisfaction of anesthetic techniques. The parental satisfaction was assessed by the PSLAS that was developed and validated by a previous study^19^. The scale consists of 20 items that measure the parents’ perception of the information, cost, concern, recommendation, anesthesia related sequelae, child’s fear and child’s discomfort. The items are rated on a 5-point Likert scale, ranging from 1 (strongly disagree) to 5 (strongly agree). The questionnaire was administered to the parents by an outcome assessor who was blinded to the group assignment.

A total of 15 items involved within the PSLAS scale were answered immediately after the procedure. While the last five items were developed to be answered on the day after the procedure as they were related to the upcoming complications associated with the anesthetic techniques.

After 10 days, the child received the treatment on the other side and parents were asked to refill the scale again regarding the other technique of anesthesia.

### Statistical analysis

The data was analyzed using SPSS software (version 26.0, IBM, USA). Descriptive statistics were utilized to summarize the demographic characteristics of the 52 participants, including age, gender, and education levels. Paired sample *t*-test was used to compare between the mean value of parental satisfaction between the two groups. Regression analysis was performed to assess the impact of independent variables (Information, Concern, Cost, Child’s fear, Child’s discomfort, recommendation and anesthesia-related sequelae) on the dependent variable, Parental Satisfaction. Beta coefficients were calculated to determine the strength and direction of these relationships. In addition, independent sample *t*-test was used to analyze the difference between the two genders and one way ANOVA was used in analyzing the difference between age groups and education levels. The level of significance was set at 0.05.

### Ethics approval and consent to participate

The study was conducted in accordance with the ethical standards outlined in the Declaration of Helsinki. Ethical approval was obtained from the Institutional Review Board at Damascus university, and a signed informed consent was obtained from all participants.

## Results

A total of 52 parents were involved in this trial. The demographic characteristics of the children and parents who participated in the study are shown in Table 1. The majority were mothers (n = 30), and aged between 31 and 40 years (n = 36). Regarding the education level, the majority of the parents involved were graduate (40% of the participants). In addition, all of the parents were with low socioeconomic status.

**Table 1:** demographic characteristics of the participants.

Characteristics	N
Age of child in years (mean ± SD)	7.5 ± 0.5
Gender of child	
Girls	22
Boys	30
Age group of parents (years)	
20–30	12
31–40	36
41–50	3
Older than 50	1
Gender of parents	
Mothers	30
Fathers	22
Level of education	
Illiterate	5
Primary	15
High school	17
Graduate	12
Postgraduate	3
Socioeconomic status index	
High	0
Medium	0
Low	52

PSLAS scale used in this study is composed of 20 items, and the answers were arranged as follows, 1: strongly disagree, 2: disagree, 3: not sure, 4: agree and 5: strongly agree. Therefore, parental satisfaction was classified into five categories; very low (0–20), low (21–40), moderate (41–60), high (61–80) and very high (81–100). Percentage, minimum score, maximum score, mean and standard deviation of parental satisfaction in both groups are summarized in Table 2. Paired sample* t*-test showed that the mean of parental satisfaction in CIA group was higher than IANB group with a statistically significant difference as shown in Table 2 also.

**Table 2:** Percentage, minimum score, maximum score, mean and standard deviation of parental satisfaction in both groups.

PSLAS	very low	Low	Moderate	High	very high	Min	Max	Mean	SD*	T-value	*P*- value
IANB group	1 (1.9%)	17 (32.7%)	22 (42.3%)	10 (19.2%)	2 (3.8%)	28	98	53.12	13.86	12.3	0.001*
CIA group	0 (0%)	7 (13.4%)	9 (17.3%)	25 (48%)	11 (21.1%)	29	100	83.06	11.77		

In this study, there were no instances where the local anesthesia step needed to be repeated for any patient. The initial administration of both the Quicksleeper 5 device and the Inferior Alveolar Nerve Block (IANB) technique provided sufficient anesthesia for the dental procedures performed. This outcome underscores the effectiveness of both techniques in achieving adequate anesthesia in pediatric patients.

During anesthesia, there was no complication to be registered. However, there was lip biting in 19 child who were anesthetized by IANB. In addition, 12 of that 19 children suffered from an ulcer on their lower lips due to the biting associated trauma.

Table 3 presents the item mean scores, which ranged in IANB group from 2.231 for item 1 “My child was not afraid of the tools used in local anesthesia.” to 4.565 for item 3 “The information that was provided to me about the technique of anesthesia was insufficient.”.

**Table 3:** Paired sample *t*-test regarding the difference between the two groups in each item.

Item	Mean of values (IANB)	Mean of values (CIA)	t-value	*P*-value
Immediately after completing the procedure				
1- My child was not afraid of the tools used in local anesthesia	2.231	4.333	3.12	0.001*
2- I think that the tools used to anesthetize my child’s tooth was painful	2.566	4.360	2.11	0.000*
3- The information that was provided to me about the technique of anesthesia was insufficient	4.565	4.260	1.32	0.09
4- All my questions were answered clearly before performing the anesthesia	4.211	4.299	1.09	0.08
5- I was afraid of complications related to the anesthetic technique	4.111	2.761	1.96	0.000*
6- I think that my child was feeling pain during the anesthesia	2.673	3.740	4.63	0.011
7- My child had no pain during the dental procedure he received after the anesthesia	2.626	4.111	0.98	0.002*
8- My child was happy and smiley after finishing the appointment	2.773	4.009	0.96	0.002*
9- When I asked my child about his feeling during the anesthesia, he/she told me that is was bad	2.560	4.001	0.88	0.001*
10- I think that my child’s experience with this anesthetic technique will encourage him/her to treat his/her teeth in the future	2.913	4.211	1.21	0.000*
11- I think that my child will be against coming to the dental clinic next time	2.733	3.987	1.34	0.013
12- I have no objection to pay extra expenses to comfort my child and to avoid hurting him/her	3.211	2.073	0.98	0.001*
13- I think that using this technique of anesthesia is considered as unnecessary welfare	3.875	2.221	1.88	0.002*
14- I think that this technique of anesthesia decreased my child’s dental fear	2.555	4.773	2.86	0.000*
15- I will not repeat my child’s experience once again for me or for my children	2.677	4.325	5.12	0.001*
One day after the anesthesia				
16- There was no lip biting to be reported after the anesthesia	3.011	4.310	4.98	0.001*
17- There was no ulcer that could complain my child after anesthesia	2.976	4.221	1.50	0.05
18- Eating after the anesthesia was annoying to my child	2.987	4.122	4.52	0.001*
19- My child did complain some disturbance from the area of anesthesia	2.654	4.299	1.91	0.02
20- I will not recommend others to use this technique of anesthesia for their children	2.677	4.289	3.11	0.000*

In CIA group, the item mean scores ranged from 2.761 for item 5 “ I was afraid of complications related to the anesthetic technique.” to 4.773 for item 14 “I think that this technique of anesthesia decreased my child’s dental fear.”.

Paired sample t-test showed that there was a significant difference between the mean PSLAS value of both groups in items 1, 2, 5, 7, 8, 9, 10, 12, 13, 14, 15, 16, 18 and 20 (*P*-value < 0.005) as shown in Table 3. All of those items showed that the satisfaction values were higher in the CIA group than in IANB group. However, CIA group showed less satisfaction value regarding the item 5 “I was afraid of complications related to the anesthetic technique.”. In addition, CIA group showed less satisfaction value also regarding the item 12 “I have no objection to pay extra expenses to comfort my child and to avoid hurting him/her.” and item 13 “ I think that using this technique of anesthesia is considered as unnecessary welfare. “.

In the regression analysis, the effect of the seven factors of the scale or the independent variables (Information, concern, cost, child’s fear, child’s discomfort, recommendation and anesthesia-related sequelae) on the dependent variable, Parental Satisfaction, was tested.

The results demonstrated that all independent variables had a statistically significant impact on Parental Satisfaction except for the ‘information’. The variables explained a substantial portion of the variance in Parental Satisfaction, demonstrating their significance with an Adjusted R-squared value of 0.554. Findings are shown in Table 4.

**Table 4:** Regression analysis of independent variables on parental satisfaction.

Independent variable	Beta Coefficient	*P*-value
Information	0.764	0.011
Concern	0.235	0.000*
Cost	0.231	0.0001*
Child’s fear	0.129	0.0002*
Child’s discomfort	0.198	0.000*
Recommendation	0.176	0.0001*
Anesthesia-related sequelae	0.276	0.0002*
Dependent variable	Adjusted R-squared	
Parental satisfaction	0.554	

*statistically significant (*P*-value < 0.001).

Regarding the effect of demographic data of parents and children, parental gender and age and education level had no significant association with the satisfaction regarding neither CIA group nor IANB group. Table 5 presents the results of independent sample *t*-test used to analyze the differences between mothers and fathers and the results of one way ANOVA in analyzing the effect of age and education levels.

**Table 5:** results of independent sample *t*-test used to analyze the difference between the fathers and mothers and the results of one way ANOVA in analyzing the effect of age and education levels.

	Mean value of satisfaction in CIA group	Test value	*P*-value	Mean value of satisfaction in IANB group	Test value	*P*-value
Gender		t -value			t -value	
Fathers	86.56	2.85	0.11	58.13	3.14	0.02
Mothers	77.46			48.11		
Age		F- ratio			F- ratio	
20–30 years	87.56	16.12	0.01	53.13	17.23	0.02
31–40 years	84.66			47.32		
41–50 years	78.56			53.11		
Older than 50 years	81.66			58.92		
Education		F- ratio			F- ratio	
Illiterate	79.16	21.13	0.12	62.92	20.98	0.22
Primary	80.96			53.11		
High school	83.04			57.52		
Graduate	85.16			43.32		
Postgraduate	86.96			48.72		

## Discussion

Local anesthesia plays a pivotal role in facilitating pain-free dental procedures and mitigating anxiety and discomfort associated with dental visits. Parental satisfaction with anesthesia techniques reflects not only the clinical efficacy of the chosen method but also the perceived level of comfort, safety, and effectiveness in alleviating their child’s pain. Effective pain management strategies can significantly impact parental perceptions of the dental experience, influencing their overall satisfaction and willingness to seek dental care for their children in the future. Additionally, clear communication and shared decision-making between dental professionals and parents regarding anesthesia options are essential in addressing parental concerns, enhancing trust, and fostering a collaborative approach to pediatric dental care. By prioritizing parental satisfaction with local anesthesia techniques, dental providers should pay more attention regarding the new computerized devices used recently in local anesthesia.

The present study employed a split-mouth randomized controlled clinical trial design to compare parental satisfaction the parental satisfaction between computerized intraosseous anesthesia (CIA) and inferior alveolar nerve block (IANB) for local anesthesia in children undergoing pulpotomy of lower second primary molars. This study adhered to CONSORT guidelines for randomized controlled clinical trials, ensuring a structured and rigorous approach to data collection and analysis.

Parental satisfaction regarding dental local anesthetic techniques had not been addressed in previous literature. Therefore, a scale composed of a Likert scale questionnaire was selected as the most appropriate instrument to detect parental satisfaction values^19^.

The scale used in this study is Parental Satisfaction of Dental Local Anesthetic Techniques Scale (PSLAS), which was developed and validated in the study of Alkhouli et al.^19^. PSLAS is composed of 20 items that are categorized into seven factors (Information, Concern, Cost, Child’s fear, Child’s discomfort, recommendation and anesthesia-related sequelae)^19^.

The results of this study rejected the null hypothesis and supported the alternative hypothesis that CIA is superior to IANB in terms of parental satisfaction. The results showed that the CIA group had significantly higher scores of PSLAS than the IANB group in the majority of the items of the PSLAS. This indicates that the parents perceived less pain, better behavior, and lower postoperative complications for their children who received CIA, as well as they recommend the use of CIA for others. Consequently, more overall satisfaction regarding CIA techniques^12^.

One possible explanation for the higher parental satisfaction with CIA is the faster onset, shorter duration, and higher success rate of the anesthesia, which may reduce the pain and discomfort of the injection and the dental treatment, as well as the risk of complications such as lip biting, hematoma, or nerve damage^13^. Another possible explanation is the shape of the handpiece of the QuickSleeper device used in the intervention group (CIA group), which is more acceptable by the children in comparison to the conventional syringes that are used with IANB technique.

On the other hand, lower values of satisfaction was resulted in the CIA group regarding only three items that are belongs to Concern and Cost. Item 5 ‘I was afraid of complications related to the anesthetic technique.’ Showed lower mean of satisfaction in CIA group. Parents expressed greater satisfaction with IANB in relation to fear from complications related to anesthesia warrants careful consideration. While Quicksleeper 5 may offer advantages in terms of efficacy and comfort, concerns regarding potential complications associated with anesthesia are understandably paramount for parents. The perceived familiarity and established track record of IANB may instill a sense of reassurance and confidence, thus influencing parental satisfaction in this specific aspect^20^.

Moreover, the study revealed a notable discrepancy in parental preferences and willingness to pay for anesthesia techniques. This can be attributed to that 71% of the parents involved in this study was with low level of education (illiterate, primary school and high school) and the whole sample was of low socioecononmic status. Specifically, parents with lower levels of education are less inclined to incur additional costs associated with new anesthetic techniques^21,22^.

The findings from the regression analysis, indicating that all factors of the scale had an effect on parental satisfaction, except for the ‘Information’ factor underscore the multifaceted nature of parental perceptions and attitudes towards dental anesthesia techniques. This comprehensive impact suggests that various factors, ranging from practical considerations to emotional concerns, collectively contribute to shaping parental satisfaction levels in this context.

The availability and quality of information provided to parents about anesthesia techniques can significantly impact their satisfaction levels^23,24^. However, comprehensive and clear communication from the dental practitioner who performed the both techniques of anesthesia about the benefits, risks, and alternatives of different anesthesia options helped parents make informed decisions aligned with their preferences and priorities.

This finding underscores the influence of socioeconomic factors and educational attainment on healthcare decision-making within the pediatric dental context. Parents with lower levels of education may face various barriers regarding choosing anesthesia techniques. The preference for IANB among this demographic may stem from practical considerations and a desire to minimize financial burden, reflecting disparities in healthcare access and decision-making autonomy^25^.

The identification of associations between parental satisfaction levels and various demographic factors adds depth to our understanding of the dynamics at play in the context of dental anesthesia preferences. The lack of significant differences in satisfaction levels between fathers and mothers suggests that gender does not play a significant role in shaping parental preferences regarding anesthesia techniques. This finding challenges traditional gender stereotypes and underscores the importance of considering individual preferences and experiences rather than making assumptions based on gender roles^26^. It also highlights the need for inclusive communication strategies that address the concerns and preferences of both mothers and fathers in the dental care setting^27^. Similarly, The potential impact of parental gender and age on satisfaction with anesthesia techniques was carefully examined in this study. Our analysis revealed no statistically significant differences in parental satisfaction based on these variables. This indicates that both mothers and fathers, as well as parents from different age groups, reported similar levels of satisfaction with the CIA and the IANB technique. The lack of variation suggests that parental satisfaction is more closely related to the specific aspects of the anesthesia experience, such as the child’s comfort and the perceived effectiveness of the anesthesia, rather than demographic factors. These findings are consistent with previous research that has shown parental satisfaction with pediatric dental care to be influenced primarily by the immediate clinical outcomes and the child’s response to treatment, rather than the demographic characteristics of the parents. Consequently, dental practitioners can focus on optimizing clinical practices and patient care strategies to enhance satisfaction across diverse parental demographics^28^.

The results of this study have important implications for the field of dental anesthesia and pediatric dentistry. First, they suggest that CIA is a viable and effective alternative to IANB for local anesthesia in children, especially for those who have fear or difficulty with conventional injections. Second, they indicate that CIA can improve the quality of care and the patient’s experience by increasing the parental satisfaction, which is a key outcome measure in pediatric dentistry. Third, they demonstrate that CIA can reduce the complications associated with local anesthetic techniques.

However, this study also has some limitations that should be acknowledged. The participants were recruited from a single centre (pediatric dentistry department at Damascus University), which may limit the generalizability and representativeness of the results. Therefore, based on the results and limitations of this study, more diverse samples should be used in future studies to increase the statistical power and the external validity of the results. In addition, one of the limitations of this study is the use of a subjective questionnaire, in which parents responded.

## Conclusion

This research paper compared the parental satisfaction of Computerized intraosseous anesthesia and inferior alveolar nerve block for local anesthesia in children undergoing pulpotomy of lower second primary molars. It was concluded that CIA was significantly superior to IANB in overall parental satisfaction. However, parental satisfaction values were lower in CIA group regarding costs and concern from complications. In addition, it was concluded that there was no difference in satisfaction levels regarding the gender, age and education level of the parents.

## Data Availability

The datasets used and/or analysed during the current study are available from the corresponding author on reasonable request.
